# Significant Correlation between Regional Tissue Oxygen Saturation and Vital Signs of Critically Ill Infants

**DOI:** 10.3389/fped.2017.00276

**Published:** 2017-12-21

**Authors:** Beri Massa-Buck, Virginia Amendola, Reagan McCloskey, Khodayar Rais-Bahrami

**Affiliations:** ^1^Department of Neonatology, Children’s National Health System, The George Washington University School of Medicine, Washington, DC, United States; ^2^Department of Biomedical Engineering, Children’s National Health System, The George Washington University School of Medicine, Washington, DC, United States

**Keywords:** near-infrared spectroscopy, neonate, vital signs, cerebral autoregulation, pressure passive index, regional tissue oxygen saturation, specific tissue oxygen saturation

## Abstract

**Background:**

Near-infrared spectroscopy (NIRS) has been used to non-invasively measure specific tissue oxygen saturation (StO_2_) continuously. Cerebral autoregulation status can be derived from NIRS and arterial blood pressure. The relationship of both cerebral and somatic StO_2_, fractional tissue oxygen extraction (FTOE), and cerebro-splanchnic oxygenation ratio (CSOR) with measured vital sign parameters for Neonatal Intensive Care Unit (NICU) patients has not been well studied.

**Objective:**

The aims of this study are to determine if significant relationships of brain and somatic StO_2_, brain and somatic FTOE, and CSOR parameters with vital signs for neonates exist and assess relationship between pressure passivity index, cerebral autoregulation, and mean blood pressure (MBP).

**Design/methods:**

Neonates weighing < 5 kg, preferentially with an arterial catheter, were enrolled in the study. FORE-SIGHT Elite (CASMedical Systems, Inc., Branford, CT, USA) cerebral and somatic NIRS sensors were placed over the abdominal right upper quadrant and right frontal-temporal area of the forehead for 24 h. Vital signs including arterial MBP were recorded simultaneously from the patients’ bedside monitor. Data were averaged into 60 s windows and analyzed using linear regression. Results were stratified by gestational age (GA), birth weight (BW), and presence of brain abnormality.

**Results:**

Data were obtained from 27 subjects (GA 22.2–42 weeks). Two subjects did not have an arterial line, thus they were not included in the MBP measurements. There were ~28,000–31,000 paired data points per comparison. Significant positive and negative correlations (*p* value < 0.0001) were noted between NIRS parameters and vital signs. When stratified by BW, there was a positive correlation between brain StO_2_ (StO_2_B) and MBP in the <1,500 g BW group (*r* = 0.193) and a negative correlation in >1,500 g group (*r* = 0.057). Brain and somatic FTOE in <1,500 g BW revealed a negative correlation with MBP (*r* = 0.172 and *r* = 0.086, respectively). In patients with an abnormal brain scan, a positive correlation was noted between StO_2_B and MBP (*r* = 0.354), and a negative correlation was noted between FTOE-B and MBP (*r* = 0.305). Generated pressure passive index plots suggested good cerebral autoregulation at low normal MBP ranges for lower weight and GA subjects.

**Conclusion:**

There is a significant correlation between cerebral and somatic StO_2_ and FTOE with measured vital sign parameters in NICU patients.

## Introduction

Critically ill infants in the Neonatal Intensive Care Unit (NICU) require constant monitoring of their vital signs *via* invasive and non-invasive measures. Continuous monitoring in the intensive care unit is necessary in evaluating clinical status fluctuations of a patient. Invasive techniques such as blood pressure monitoring *via* an umbilical arterial catheter or a peripheral arterial line may be the gold standard, but they do pose the risk of bleeding, infection, thrombosis, or vasospasm (1).

Non-invasive monitoring *via* continuous cardiorespiratory monitors and pulse oximetry is most common. It allows for detection of heart rate, respiratory rate, and arterial oxygen saturation. Near-infrared spectroscopy (NIRS) allows for specific tissue oxygen saturation (StO_2_) measurement continuously and non-invasively by measuring chromophores in the body such as hemoglobin, myoglobin, and cytochrome aa3 (2–4). It reflects tissue oxygen supply and demand regionally allowing one to calculate fractional tissue oxygen extraction (FTOE) and cerebro-splanchnic oxygenation ratio (CSOR) (5, 6). In addition to traditional non-invasive monitoring, NIRS provides monitoring of perfusion altering neonatal diseases including but not limited to necrotizing enterocolitis, intraventricular hemorrhage, and periventricular leukomalacia (7).

Using NIRS and arterial blood pressure to measure cerebral autoregulation has been well studied. Autoregulation status is determined to be intact when there is negative relationship between cerebral perfusion pressure or arterial blood pressure and NIRS parameters. There are many different NIRS methodologies to assess this measurement as reviewed by Kooi et al (8). Trending cerebral and/or somatic NIRS under various clinical circumstances has also been well studied (2, 9–13). However, the comparison of cerebral and somatic NIRS parameters with measured vital signs in critically ill infants in the NICU has not been well studied.

In this prospective observational study, we sought to determine if there are significant relationships between NIRS cerebral and somatic StO_2_, brain and somatic FTOE, and CSOR parameters with vital signs for neonates. Using the data we collected, we also calculated a pressure passive index (PPI) of cerebral perfusion to determine the relationship between pressure passivity, cerebral autoregulation, and mean blood pressure (MBP).

## Materials and Methods

This study was approved by the Institutional Review Board (IRB) at Children’s National with waiver of documentation of informed consent. Infants were recruited from the 54-bed NICU at Children’s National Health System. After verbal parental agreement for voluntary participation, a study information sheet was provided to the parents of infants admitted to the NICU between July 15, 2015 and December 17, 2016, preferentially with an umbilical or peripheral arterial line. Infants with congenital heart disease, history of abdominal surgeries, and/or surgical necrotizing enterocolitis were excluded. Patients’ demographic and clinical data including gender, race, birth weight (BW), weight at the time of the study, gestational age (GA), age at the time of study, total combined age (GA + age at the time of study), primary diagnosis, brain examination by ultrasound or MRI, length of hospital stay, and morbidity were documented. Abnormal brain scan results included presence of intraventricular hemorrhage, cerebellar hemorrhage, encephalopathy, or structural brain anomalies.

Twenty-seven infants born between 22 and 42 weeks gestation were enrolled in this study. Subjects were monitored with a pulse oximeter, peripheral or umbilical arterial line, and FORE-SIGHT Elite (CASMedical Systems, Inc., Branford, CT, USA) cerebral and somatic NIRS sensors. NIRS sensors were placed over the right upper anterior abdominal wall for StO_2_S measurements and right frontal-temporal area of the forehead (StO_2_B measurements) for 24 h. Arterial lines were placed either prior to transfer to or at Children’s National NICU for patients’ clinical management only. Pulse oximetry (SpO_2_) was used to obtain arterial oxygen saturations (SaO_2_). Remaining vital signs (heart rate, respiratory rate, and continuous blood pressure from the umbilical or peripheral arterial line) data were obtained from the patient’s bedside monitor. The data were recorded simultaneously every 2 s on a laptop computer. NIRS monitoring did not interfere with patient care and was allowed to be stopped at the option of the caregiver or parents.

### Data Analysis

Due to numerous data points, the data were averaged into 60-s windows and combined for all subjects for the respective parameter of interest, such as mean value for brain StO_2_ (StO_2_B), somatic StO_2_ (StO_2_S), SpO_2_ and for vital signs, blood pressure (diastolic, mean, and systolic), heart rate, and respiratory rate were calculated for each 60-s binned window. Mean StO_2_B and StO_2_S data points were omitted for mean arterial blood pressures beyond physiologic range (i.e., >100.) Brain fractional tissue oxygenation extraction (FTOE-B) was calculated by the following equation: $\frac{{\text{SpO}}_{2}-{\text{StO}}_{2}\text{B}}{{\text{SpO}}_{2}}$, and Somatic FTOE (FTOE-S) was calculated by $\frac{{\text{SpO}}_{2}-{\text{StO}}_{2}\text{S}}{{\text{SpO}}_{2}}$ (6). Cerebro-splanchnic oxygenation ratio (CSOR) was calculated by the following equation: $\frac{{\text{StO}}_{2}\text{S}}{{\text{StO}}_{2}\text{B}}$ (5). Using linear regression, StO_2_B, StO_2_S, SpO_2_, FTOE-B, FTOE-S, and CSOR were correlated with vital sign parameters and age indices to determine a correlation coefficient “*r*” to determine any relationships.

### Pressure Passive Cerebral Perfusion Index

A PPI of cerebral perfusion as a function of MBP was calculated and binned in 5 mmHg epochs for MBP for each subject using coherence analysis with a prototype cerebral autoregulation algorithm (14–16). StO_2_B and MBP sampled at 2 s were used for the analysis. PPIs derived from coherence values were calculated in the frequency band of 0.00167–0.15 Hz (6.67 s to 20 min sampling window) and binned every 30 s for the respective MBP epoch if a change in MBP was detected. Once completed, PPI vs MBP epochs were plotted for each subject and then averaged together per MBP epoch in several subject groupings by BW/GA, current weight/total age, and brain scan results (normal and abnormal) to determine any differences. A high PPI is indicative of poor cerebral autoregulation function for a given MBP range.

## Results

Between July 15, 2015 and December 17, 2016, 27 infants (18 males and 9 females) were enrolled in this study. The median GA was 31.2 weeks (range: 22.2–42 weeks), and median BW was 1,266 g (range: 480–4,170 g). Median length of stay was 49 days (range: 7–189 days). Two patients had confirmed cases of sepsis, 2 patients had NEC, 11 patients had IVH, and 3 patients died before discharge. Two subjects did not have an arterial line, and thus they were not included in the mean arterial pressure measurements. Table 1 summarizes patients’ demographic information.

**Table 1:** Patient demographics, *n* = 27, male = 18 (67%).

	Median (range)
Gestational age at delivery (weeks)	31.2 (22.2–42)
Birth weight (g)	1,266 (480–4,170)
Weight at time of study (g)	2,210 (530–4,030)
Age at the time of study (days)	6 (0–143)
Length of hospital stay (days)	49 (7–189)
Number of patients with:	
Confirmed sepsis	2^a^
NEC	2^a^
Brain abnormalities	16^a^
Mortality	7.4%

*^a^Total number of patients*.

Table 2 shows the linear regression Pearson correlation coefficient (*r*) values of StO_2_B, StO_2_S, SpO_2_, FTOE-B, FTOE-S, and CSOR compared to blood pressure, heart rate, respiration rate, and subject age indices for all subject data combined using the 60-s binned data. Although the correlations were weak, all comparisons were statically significant (*p* < 0.0001). The oxygen saturation parameters (StO_2_B, StO_2_S, and SpO_2_) showed positive correlation with blood pressure and most age indices and a negative correlation with heart rate. FTOE-B and FTOE-S showed negative correlation with blood pressure and age indices. CSOR did not correlate as highly as the other oxygenation indices with vital signs and age indices. Respiration rate showed low correlation with the oxygenation indices.

**Table 2:** Linear regression Pearson correlation coefficient (*r*) values of brain specific tissue oxygen saturation (StO_2_B), somatic StO_2_ (StO_2_S), specific tissue oxygen saturation (SpO_2_), brain fractional tissue oxygen extraction (FTOE-B), somatic FTOE (FTOE-S), and CSOR compared to blood pressure (diastolic, mean, and systolic), heart rate, respiration rate, and subject age indices for all subject data combined using the 60-s binned data.

Pearson regression correlation coefficient (*r*)	StO_2_B	StO_2_S	SpO_2_	FTOE-B	FTOE-S	CSOR
ABP (diastolic)	0.285	0.175	0.277	−0.224	−0.116	0.044
ABP (mean)	0.266	0.159	0.259	−0.213	−0.113	0.049
ABP (systolic)	0.22	0.126	0.216	−0.183	−0.101	0.054
HR	−0.171	−0.148	−0.218	0.089	0.079	−0.058
RR	−0.02	0.056	0.048	0.041	−0.038	0.075
Age	−0.081	−0.199	−0.021	0.076	0.201	−0.166
Gestational age (GA) at birth	0.364	0.277	0.348	−0.285	−0.213	0.088
Total age (GA + age)	0.322	0.151	0.336	−0.238	−0.076	−0.018
Birth weight	0.348	0.285	0.336	−0.26	−0.204	0.098
Current weight	0.355	0.229	0.324	−0.27	−0.136	0.031

*There were ~29,000–39,000 paired data points per comparison where all comparisons were statically significant (*p* < 0.0001)*.

*All statistically significant (*p* < 0.0001)*.

*ABP, arterial blood pressure*.

**Figure 1:**
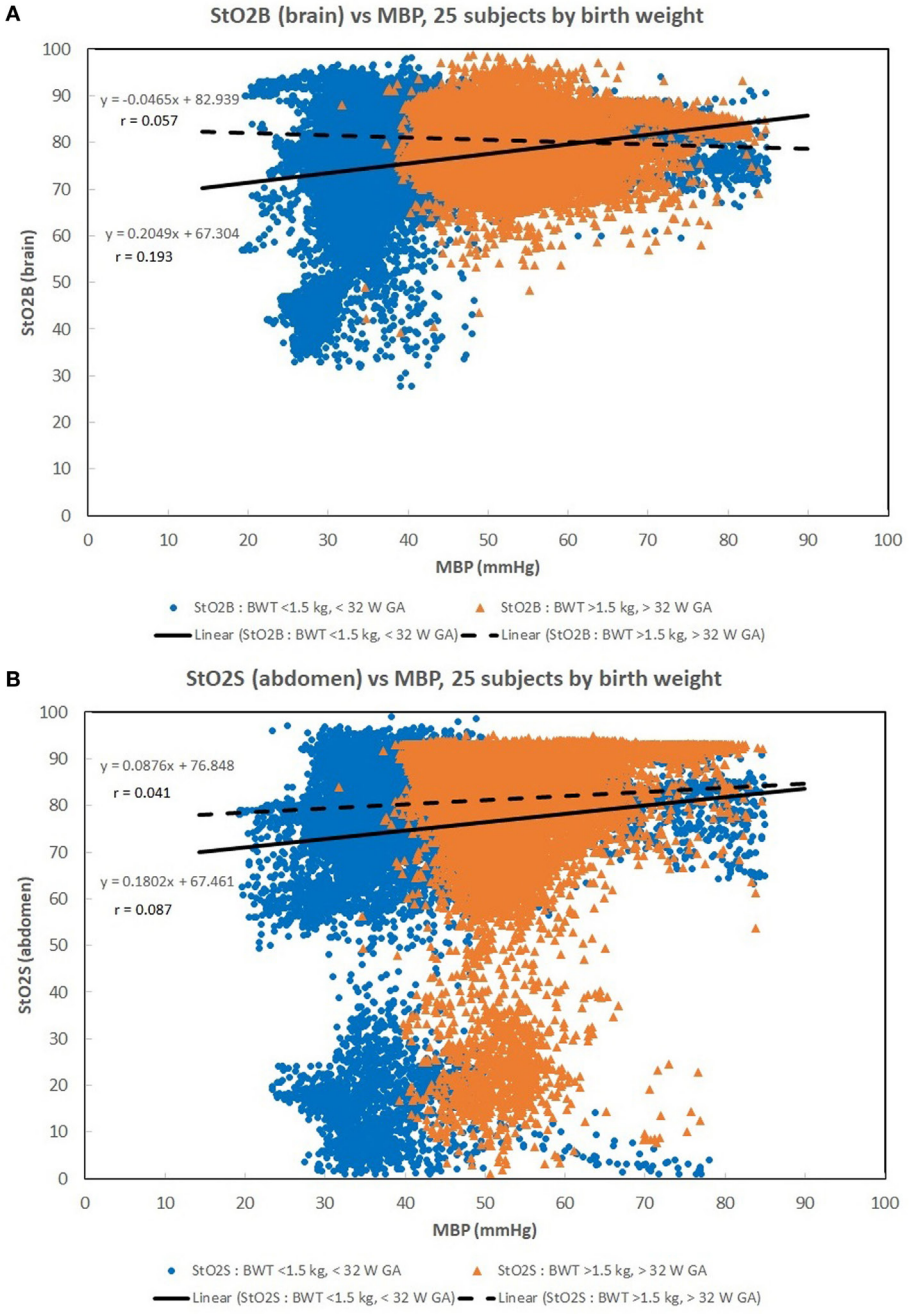

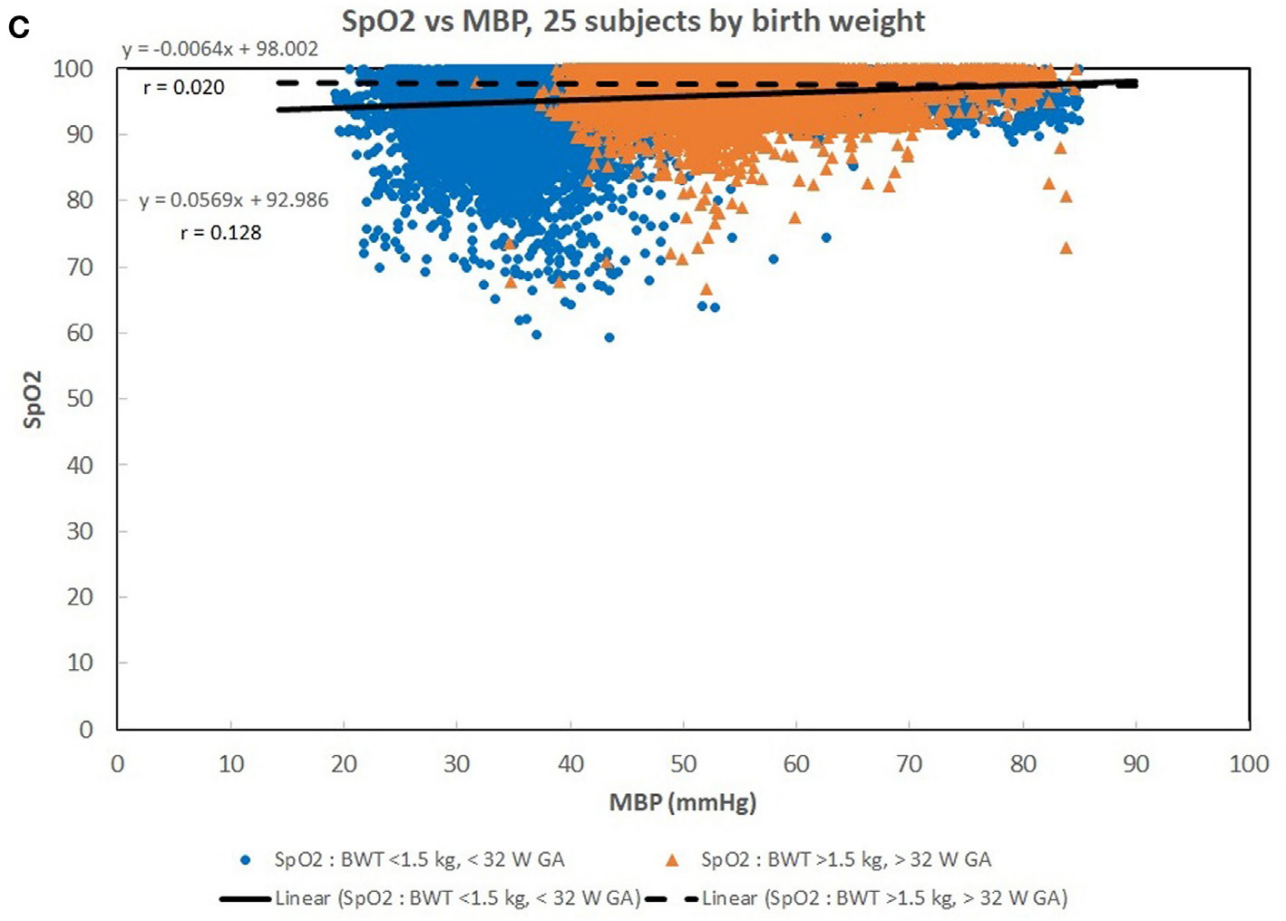
**(A–C)** Scatter plots of the oxygen saturation parameters [brain specific tissue oxygen saturation (StO_2_B), somatic StO_2_ (StO_2_S), and pulse oximetry (SpO_2_)] vs mean blood pressure (MBP) with subjects divided into two groups: low birth weight [LBW, BW < 1.5 kg/gestational age (GA) < 32 weeks (*n* = 13)] vs moderate birth weight [BW > 1.5 kg/GA > 32 weeks (*n* = 12)]. The LBW group showed a higher linear regression correlation (*r*) to the oxygen saturation parameters, where the strongest correlation was with StO_2_B. There were 33,000–35,000 valid (60 s binned) paired data points to create these scatter plots.

**Figure 2:**
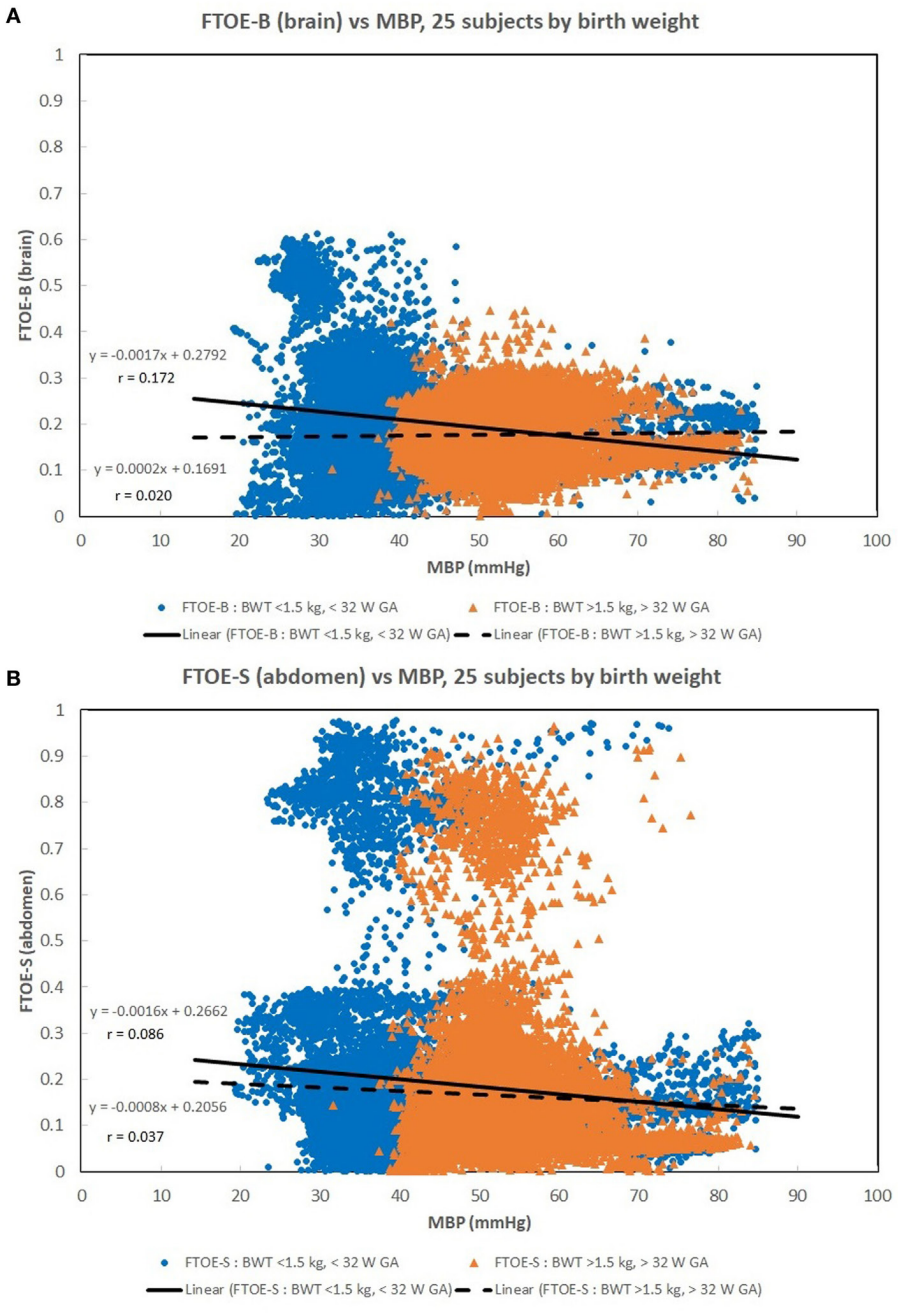

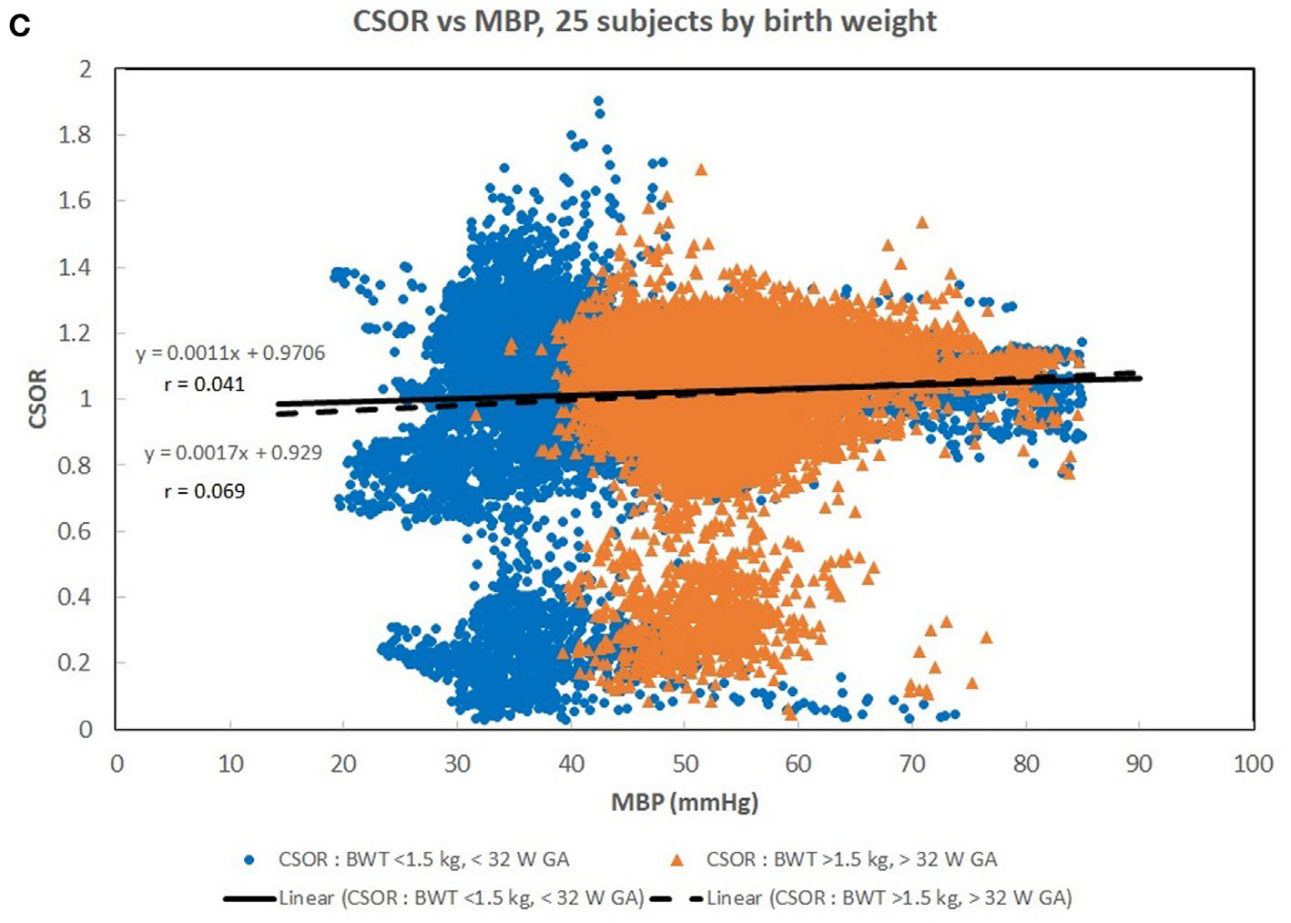
**(A–C)** Scatter plots of the oxygenation indices [brain fractional tissue oxygen extraction (FTOE-B), somatic fractional tissue oxygen extraction (FTOE-S), and CSOR] vs mean blood pressure (MBP) with subjects divided into low birth weight (LBW) and moderate birth weight (MBW) groups like that of Figure 1. The LBW group showed a higher linear regression negative correlation (*r*) to FTOE-B and FTOE-S, where the strongest correlation was with FTOE-B. CSOR weakly correlated to MBP, with little differences between LBW and MBW groups. There were 28,000–31,000 valid (60 s binned) paired data points to create these scatter plots.

**Figure 3:**
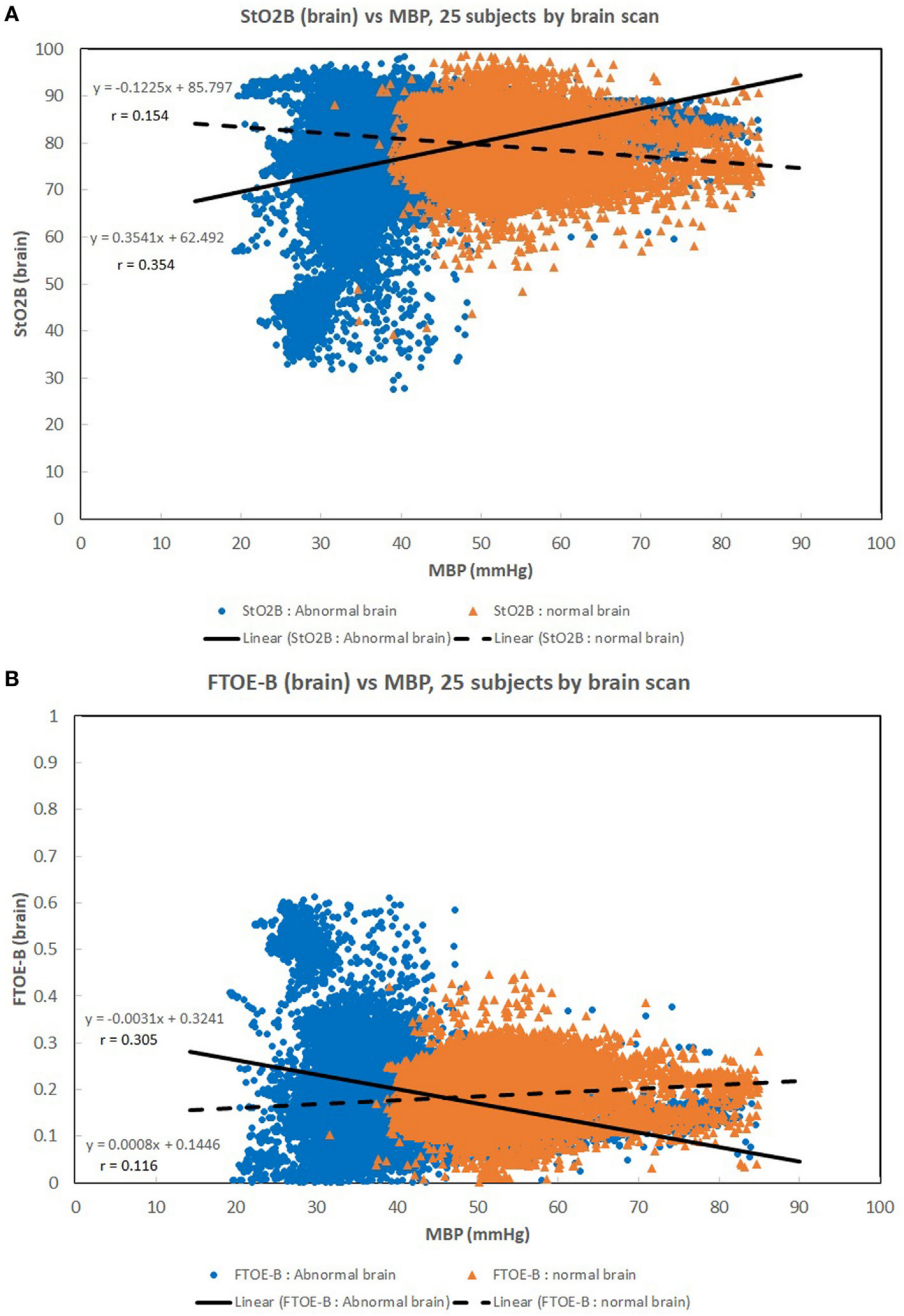
**(A,B)** Scatter plots of brain specific tissue oxygen saturation (StO_2_B) and brain fractional tissue oxygen extraction (FTOE-B) vs mean blood pressure (MBP) with subjects grouped by brain scan results: normal brain (*n* = 10) and abnormal brain (*n* = 15). Both the linear regression slopes and correlation (*r*) values were greater than the previous low birth weight (LBW) group for StO_2_B and FTOE-B vs MBP of the previous figures. Note that 11 of 15 subjects with brain abnormalities were in the LBW group.

Figures 1A–C show scatter plots and linear regression lines of the oxygen saturation parameters (StO_2_B, StO_2_S, and SpO_2_) vs MBP of subjects divided into two groups: low birth weight [LBW; BW < 1.5 kg/GA < 32 weeks (*n* = 13)] vs moderate birth weight [MBW; BW > 1.5 kg/GA > 32 weeks (*n* = 12)]. The LBW group showed a higher correlation (Pearson *r*) with the oxygen saturation parameters, where the strongest correlation was with StO_2_B. Figures 2A–C show scatter plots and linear regression lines of the oxygenation indices (FTOE-B, FTOE-S, and CSOR) vs MBP with subjects divided into the same two groups LBW and MBW. The LBW group showed a higher negative correlation (Pearson *r*) with FTOE-B and FTOE-S, where the strongest correlation was with FTOE-B. CSOR showed low correlation with MBP, with little differences between LBW and MBW. Figures 3A,B show the scatter plots and linear regression lines of StO_2_B and FTOE-B vs MBP with subjects grouped by brain scan results: normal brain (*n* = 10) and abnormal brain (*n* = 15). Both the linear regression slopes and *r* values were higher than the previous LBW group for StO_2_B and FTOE-B vs MBP. Note that 11 of 15 subjects with brain abnormalities were in the LBW group.

**Figure 4:**
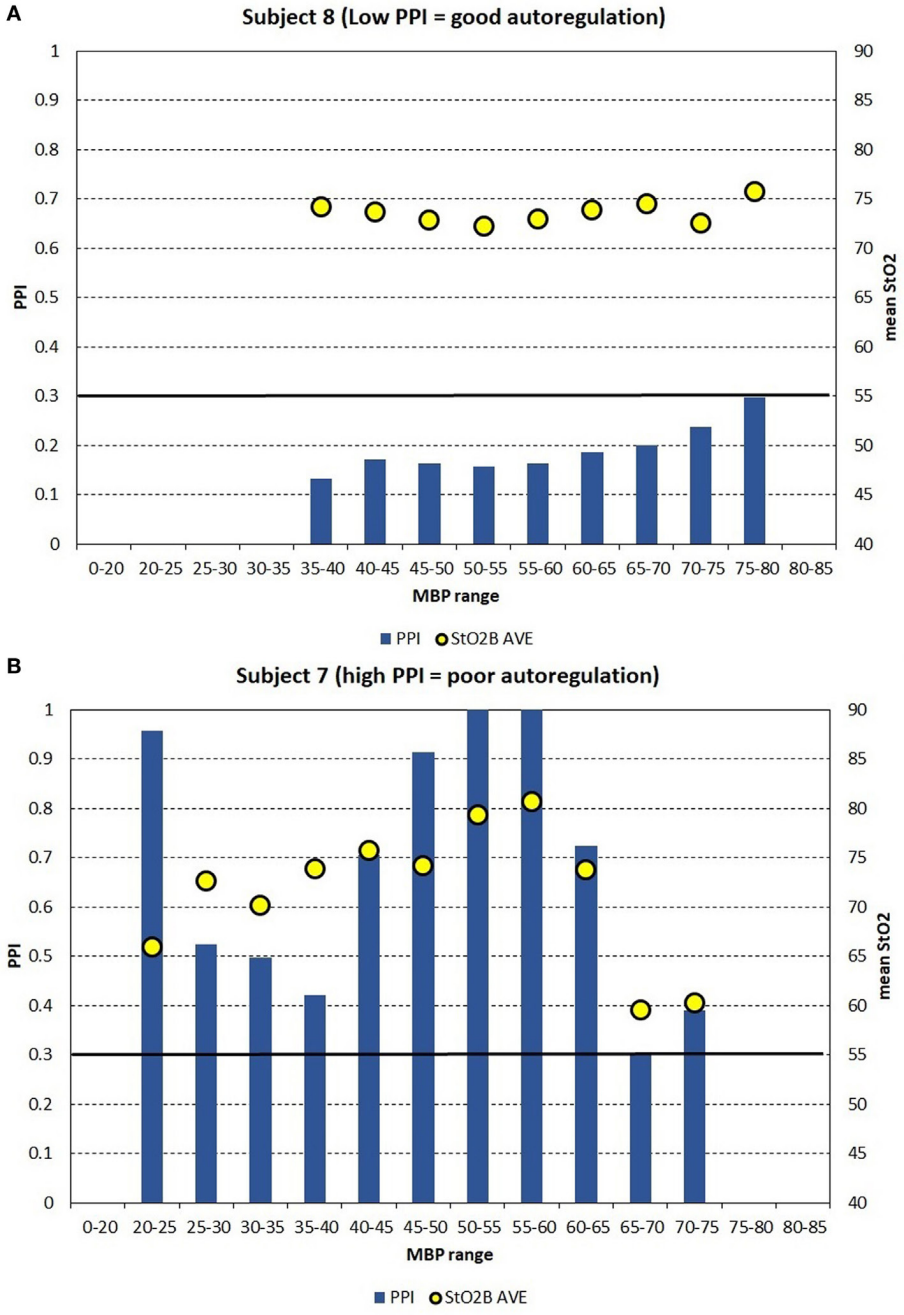

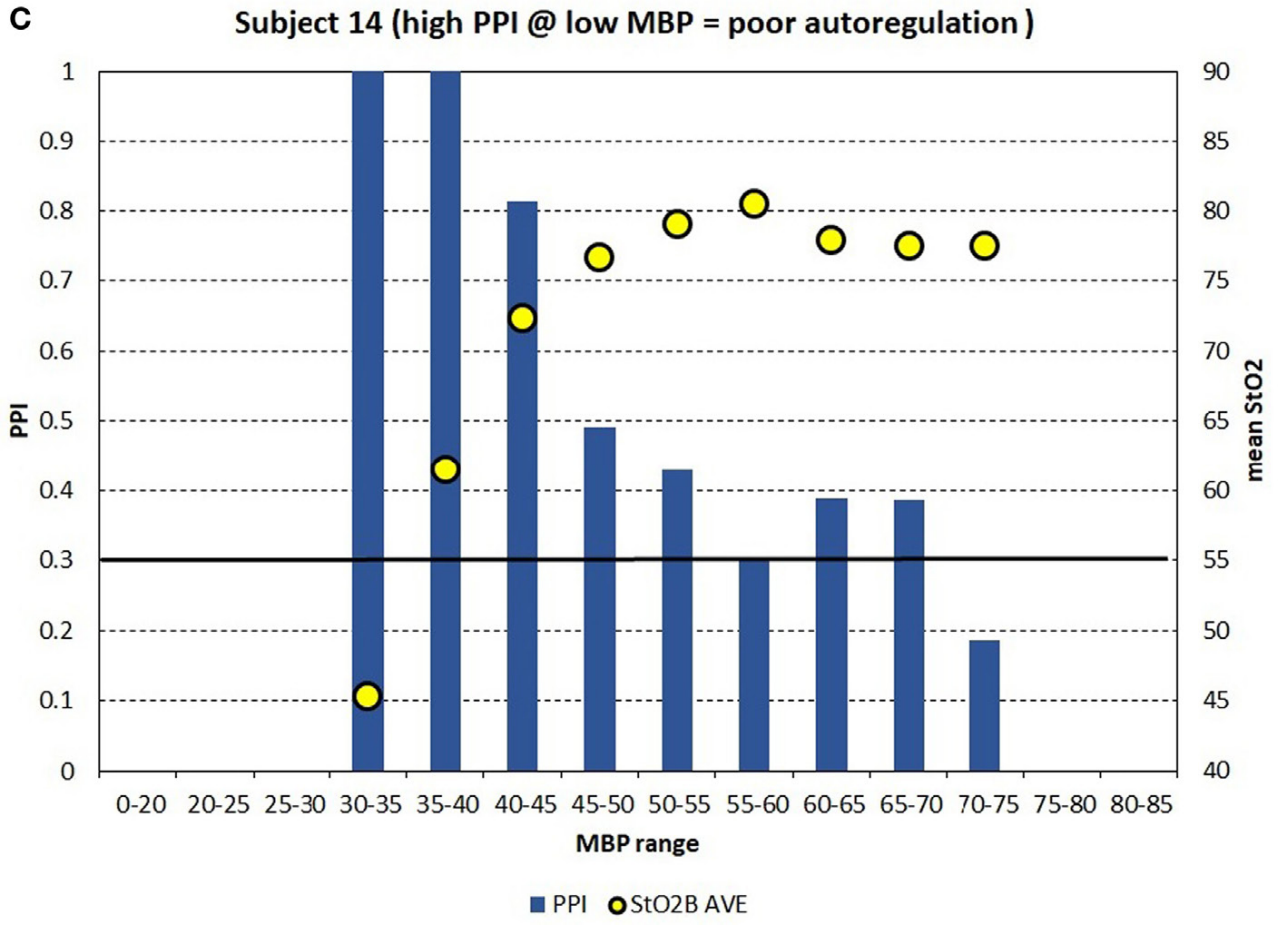
**(A–C)** For case study demonstration purposes, the pressure passive index (PPI) is binned into discrete mean blood pressure (MBP) ranges to create PPI profile plots for three subjects, which can be interpretable to autoregulation function. **(A)** Shows subject data where PPI is low for the MBP ranges measured, where it is believed that autoregulation function is good. **(B)** Shows subject data where PPI is generally high for the MBP ranges measured, where it is believed that autoregulation function is poor. **(C)** Shows subject data where PPI is high for low MBP, and PPI is moderate for higher MBP, where it is believed that autoregulation function is poor for low MBP and moderate for higher MBP. The arbitrary black line at PPI = 0.30 is hypothesized to be a transition point where autoregulation function transits gradually from poor to good.

Figures 4A–C show the PPI vs MBP profile plots of three subjects to demonstrate three examples on how PPI profile plots can be interpretable to autoregulation function. Subject 8 was a full-term appropriate for gestational age (AGA) infant without any cerebral injury. Figure 4A shows subject data where PPI is low for the MBP ranges measured, where it is believed that autoregulation function is good. Subject 7 was a preterm infant with very LBW and had cerebral injury. Figure 4B shows subject data where PPI is generally high for the MBP ranges measured, where it is believed that autoregulation function is poor. Subject 14 was an acutely ill full-term AGA infant with respiratory failure secondary to meconium aspiration, pneumothorax, and sepsis. Figure 4C shows subject data where PPI is high for low MBP, and PPI is moderate for higher MBP, where it is believed that autoregulation function is poor for low MBP and moderate for higher MBP. It is likely that autoregulation function is gradual and not a binary “good/poor” as PPI increases. During good autoregulation function, low PPI values per MBP epochs appear to be similar in values, giving a flat appearance, as PPI is always >0. If the lower blood pressure autoregulation inflection point is crossed, resulting in decreased autoregulation function, PPI will increase with decreasing MBP. For these PPI profile plots, the mean StO_2_B values are shown for each MBP epoch. StO_2_B may decrease as PPI increases for low MBP values, while StO_2_B can become variable with increased PPI in higher MBP ranges. If the upper blood pressure autoregulation inflection point is crossed, resulting in decreased autoregulation function, StO_2_B may increase due to excessive cerebral perfusion.

**Figure 5:**
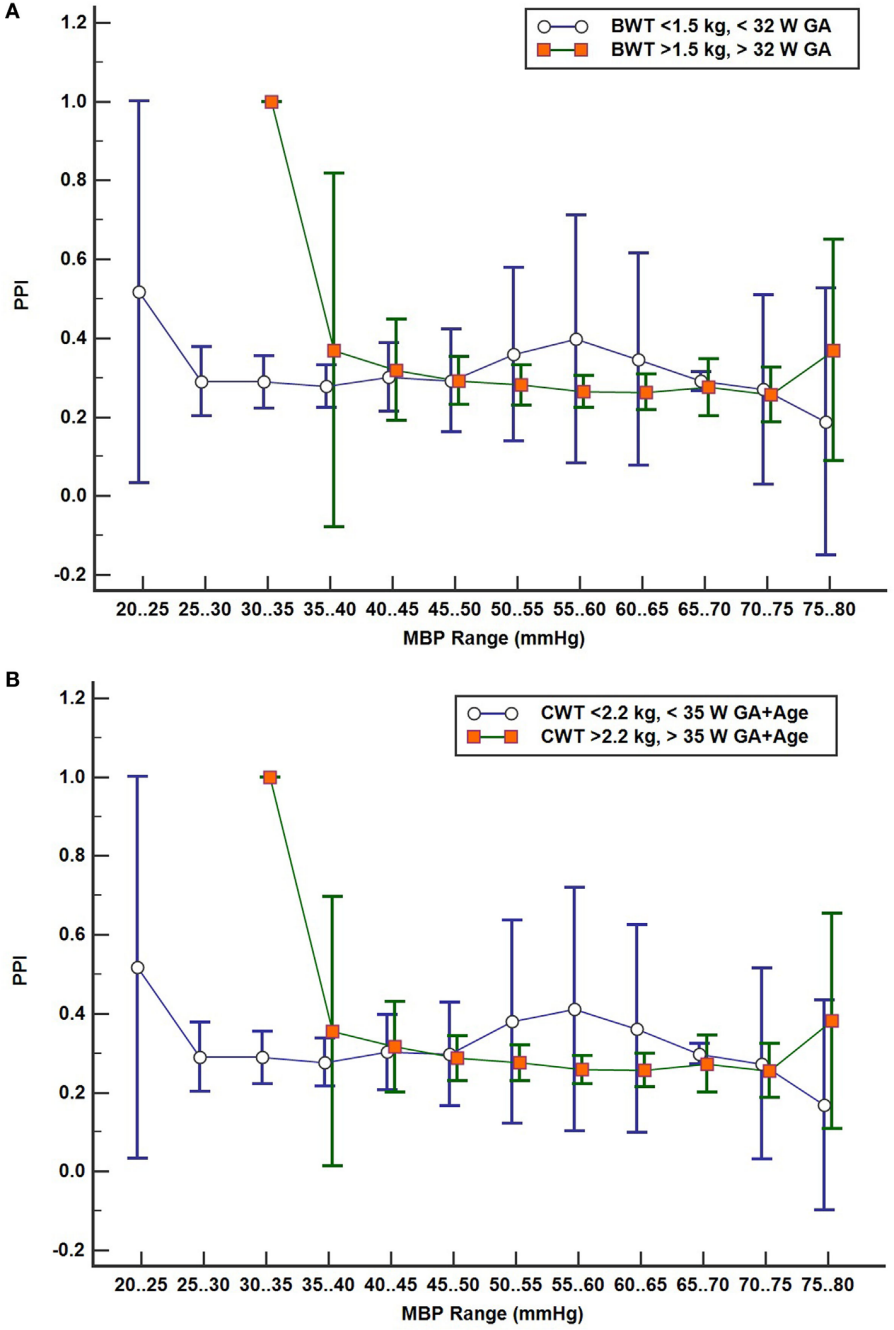

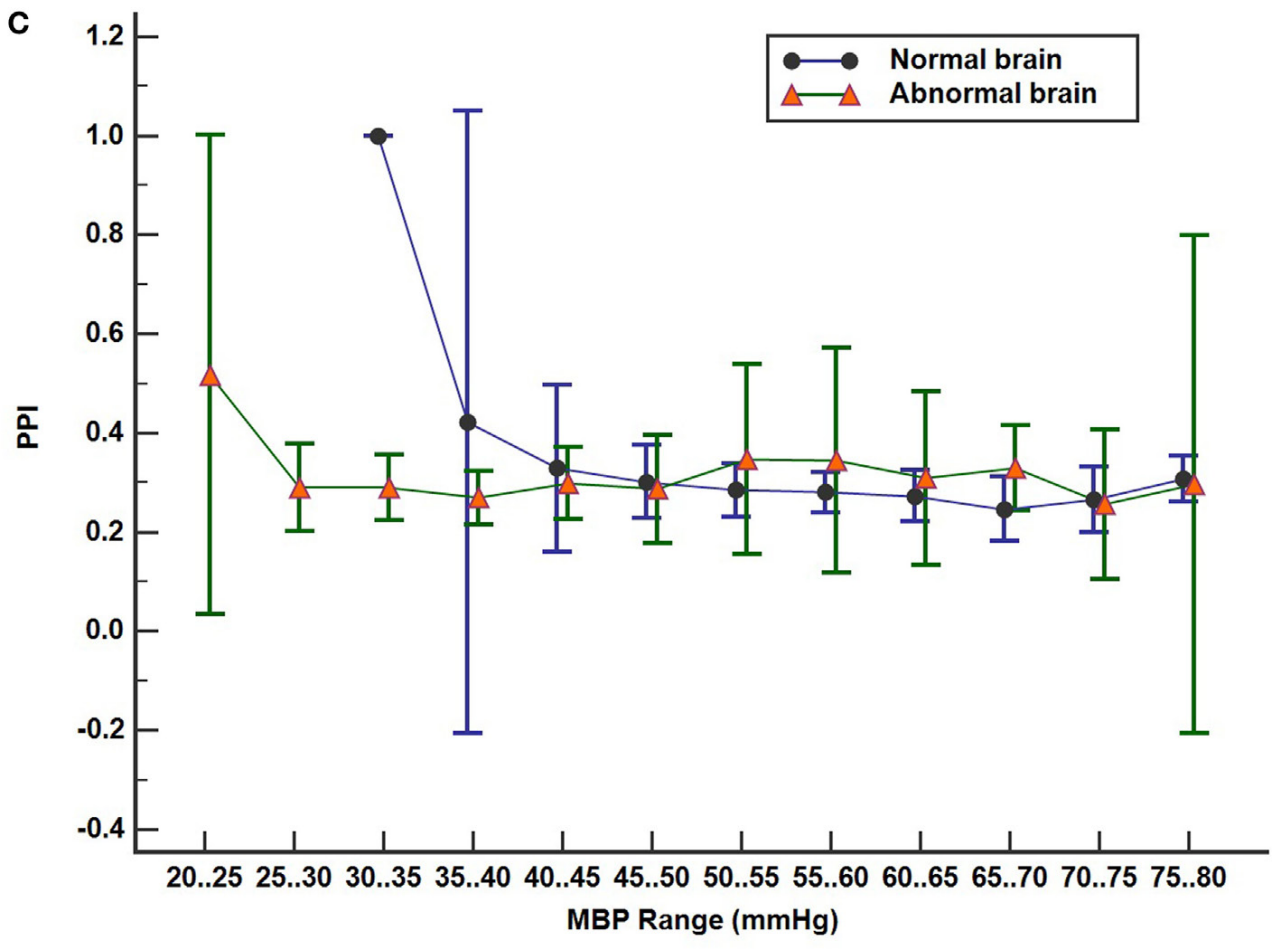
**(A–C)** Pressure passive index (PPI) profile plots with subject values binned in discrete mean blood pressure (MBP) ranges by two groups with 95% confidence interval for mean values: **(A)** by birth weight/gestational age (GA) groups low birth weight (*n* = 13) vs moderate birth weight (*n* = 12), **(B)** by current weight and total age groups LCW (*n* = 12) and MCW (*n* = 13), and **(C)** brain scan results, normal (*n* = 10) and abnormal brain (*n* = 15).

Figure 5A shows the PPI profile plot values combined in two subject groups: LBW and MBW like before. The mean PPI and 95% confidence intervals are shown for the LBW and MBW groups for each MBP epoch. PPI increases at a lower MBP for the LBW group compared to the MBW group, suggesting that the lower blood pressure autoregulation inflection point is lower for the LBW group. PPI appears to remain low for a lower range of MBP values for the LBW group where no data were recorded in this range for the MBW group. Figure 5B shows similar results, with subjects divided into two groups by current weight and total age (GA + age) at the time of study: LCW [current weight < 2.2 kg/total age < 35 weeks (*n* = 12)] vs MCW [current weight > 2.2 kg/total age > 35 weeks (*n* = 13)]. The plots look similar as the LBW and LCW groups were the same subjects except for one subject. Figure 5C shows the PPI profile plot for subjects grouped by brain scan results: normal brain (*n* = 10) and abnormal brain (*n* = 15). Other comparative analyses were limited by insufficient group sizing.

## Discussion

This study sought to determine any relationship between NIRS cerebral and somatic StO_2_, cerebral and somatic FTOE, and CSOR parameters with vital signs for neonates admitted to Children’s National NICU with various levels of clinical status severity.

Normal range arterial blood pressures are expected to provide adequate oxygenated blood to target organs. Acceptable SpO_2_ levels of 85–100% were maintained for a wide range of MBP in infants weighing >1.5 kg and >32 weeks GA compared to the LBW group. As anticipated, lower MBP allowed for normal SpO_2_ levels in the LBW group, as this subgroup of patients has a lower normal range of blood pressures compared to the MBW group.

Maintaining a normal range of mean arterial blood pressure has been emphasized in care of neonates to ensure adequate cerebral perfusion and prevent cerebral injury. The ability of cerebral vascular smooth muscle to respond to changes in systemic pressure is one mechanism that is responsible for cerebral autoregulation (17). A rise in MAP should lead to vasoconstriction with reduction of cerebral blood volume in a cerebrovascular pressure reactive system, and if defective, cerebral blood volume will increase passively (18). Due to the ability to provide continuous measurement of cerebral StO_2_ and MAP, NIRS can serve as a surrogate for cerebral blood flow and possibly assess the integrity of cerebral circulation (15, 16, 19, 20). A positive correlation was noted in Figure 1A between StO_2_B and MBP in the LBW group, and a negative correlation was seen in the MBW. Hypotension was associated with a lower StOB in the LBW group vs MBW group. Studies have shown that a negative to no correlation between mean arterial pressure and intracranial pressure, in this case MBP and NIRS as a surrogate of cerebral blood flow, can be interpreted as good vasoreactivity (21, 22). It can be inferred that the positive correlation witnessed between StOB and MBP in the LBW is due to poor vasoreactivity secondary to prematurity or cerebral injury.

A similar trend was observed in StOB and FTOE-B vs MBP with subjects grouped by presence or absence of brain abnormality, which suggests the presence of pressure passivity. A negative correlation was observed between FTOE-B and MBP. As MBP increases thus increasing oxygen delivery, FTOE-B decreases possibly to keep oxygen consumption at the cerebral tissue constant. This form of autoregulation is disrupted in infants with IVH as previously described in other studies and demonstrated in our study of the LBW group and abnormal brain group vs MBP (16). This observation is further supported by the case studies of subjects 7, 8, and 14.

Pressure reactivity is also observed during the lower range of MBP values for the LBW and LCW groups compared to MBW and MCW groups. Low PPI suggests good cerebral autoregulation at lower MBP ranges for subjects with lower weight, GA, and total age. Abnormal brain subjects also show this trend, but 11 of 15 subjects with brain abnormalities were in the LBW group.

Somatic StO_2_ vs MBP had a positive correlation for LBW and MBW. In Figures 1B and 2B, there appears to be two fields which are due to a few subjects with a low StO_2_S. However, the regression analysis is mostly affected by the high density of data points concentrated on the higher values. The positive correlation observed between StO_2_S and MBP is expected as arterial pressures increase, more oxygenated blood is provided to end target organs. Of note, for some subjects, StO_2_S decreased when arterial blood pressure was less than 75 mmHg while cerebral StO_2_ was maintained between arterial blood pressures of 50–75 mmHg. This event was thought to be secondary to preservation of perfusion to essential organs over splanchnic perfusion during hypotensive episodes (5, 23, 24).

Our study has some limitations. Although the sample size provided us with numerous data points, the majority of patients were relatively stable during the period of tissue-specific oxygenation monitoring. Placing a neonate on the monitors during critically ill periods was difficult as the patient’s moment to moment stability relied on minimal disturbances. For subjects that are relatively healthy, variations of oximetry and vital sign parameters do not expect to vary much, resulting in low correlations with each other. More subjects with varying range of disease states are needed for correlation of oximetry vs vital sign parameters to strengthen the mean and ranges of NIRS cerebral and somatic StO_2_ values. There are limitations with NIRS monitoring such as the limited area that is measured by the monitor, which is only tissue saturations directly underneath the sensor. If a larger surface area was monitored, more alterations of StO_2_ may have been detected due to increased measurement of regional tissue saturations (13). Finally, accessing patients’ arterial lines for clinical care such as blood draws can cause a spike in the arterial blood pressure thus providing a falsely elevated blood pressure reading. These readings, in addition to recorded readings when sensor strength was not optimal (i.e., sensor not flush against patient skin) may have produced outliers, thus further weakening the correlation.

In summary, we have shown that there is a significant relationship between cerebral and somatic StO_2_ to measured vital signs parameters for NICU patients, and cerebral autoregulation may be present in lower ranges of MBP values for LBW infants compared to MBW infants. A significant correlation between StO_2_ and measured vital sign parameters was present. Although the correlations are weak, this may be a reflection of the lack of heterogeneity of the subjects enrolled in this study and/or fluctuation of vital sign parameters seen in neonates. Overall, the assessment of the correlation should be used in clinical context with the patient.

## Ethics Statement

This study was carried out in accordance with the recommendations of the Institutional Review Board (IRB) at Children’s National. Parents of all the patients in the study, as our patients are neonates and unable to agree to participate in the study, gave verbal agreement for voluntary participation in the study. Parents could also refuse to participate in the study. A study information sheet was provided to the parents of infants who participated in the study. Required written consent for participation in this study was waived by the IRB. The NIRS system is considered a non-significant risk device and is used routinely in the clinical care of many patients in the NICU. This study was observational only and did not affect the care provided to the neonates.

## Author Contributions

BM-B identified and enrolled subjects, collected and analyzed data, drafted the initial manuscript, and approved the final manuscript and is accountable for all aspects of the manuscript. VA and RM determined the conception and design of data acquisition, reviewed and revised the manuscript, approved the final manuscript, and are accountable for all aspects of the manuscript. KR-B identified and enrolled subjects, collected and analyzed data, reviewed and revised the manuscript, approved the final manuscript, and is accountable for all aspects of the manuscript.

## Conflict of Interest Statement

The authors have no financial interests in the product discussed in this manuscript. Study NIRS monitor and sensors were supplied by CASMedical Systems, Inc. (Branford, CT, USA).
